# Differential stress response mechanisms in right and left ventricles

**Published:** 2016

**Authors:** Makhosazane Zungu-Edmondson, Yuichiro J. Suzuki

**Affiliations:** Department of Pharmacology and Physiology, Georgetown University Medical Center, Washington, DC 20057

**Keywords:** Ischemia/reperfusion, Left ventricle, Pulmonary hypertension, Right heart failure, Right ventricle

## Abstract

Right ventricular (RV) failure is the major cause of death among patients with pulmonary hypertension. However, differences between the RV and left ventricle (LV) of the adult heart have not been defined, despite myocytes from these two ventricles originate from different progenitor cells. The lack of such knowledge interferes with developing therapeutic strategies to protect the RV. The goal of this study was to identify possible differences between stress responses in the RV and LV free walls of adult rats. We found that levels of angiogenesis and autophagy/mitophagy proteins are higher in the LV than in the RV. Thus, the LV may be more resistant to stress-induced damage. To test this, isolated rat hearts were subjected to biventricular working heart perfusion and ischemia/reperfusion (I/R) injury. However, I/R was found to cause apoptosis in both LV and RV to a similar extent. One mechanism of cardiac apoptosis involves downregulation of GATA4 transcription factor that controls gene transcription of anti-apoptotic Bcl-xL. Interestingly, only in the RV, I/R caused downregulation of GATA4 and Bcl-xL, suggesting that mechanisms of apoptosis may be different between the two ventricles. Levels of tropomyosin and troponin T were also found to be decreased in response to I/R only in the RV, but not in the LV. Downregulation of the GATA4/Bcl-xL axis and the reduction of tropomyosin and troponin T are RV-specific events that occur in response to stress. This information may be useful for designing RV-specific therapeutic strategies to treat RV failure in pulmonary hypertension patients.

## Introduction

The pathophysiology of the left ventricle (LV) is well understood and has been considered to represent that of the heart, in general. However, despite the right heart failure is the major cause of death among patients with pulmonary hypertension, the right ventricle (RV) has not been well studied^1^. Apparent mechanisms of heart failure in the right and left sides of the heart, in response to pulmonary and systemic hypertensions, respectively, are different. Concentric hypertrophy of the LV transitions to LV dilation with eccentric cardiac hypertrophy and thinning of the LV wall. By contrast, the failed RVs in cor pulmonale have the structure of the concentrically hypertrophied RV^2,3^. It is unclear if therapies that are designed to treat LV dysfunctions benefit the RV. Thus, understanding the RV biology should help developing new therapeutic strategies for cardiovascular diseases, in particular, pulmonary hypertension.

LV and RV myocytes are developed from different precursors. Cells in the first heart field (primary heart field) contribute to the formation of the LV myocardium, whereas cells in the second heart field (anterior heart field) contribute to form the RV myocardium^4,5^. Also, the RV is subjected to pumping the blood against wide-range of pressure (~100 mmHg in utero and ~10 mmHg after birth). Thus, the biology of adult RV is expected to be different from that of the LV. However, the overall gene expression patterns of the adult RV and LV free walls are remarkably similar. Subtle differences between the two ventricles may be important for developing therapeutic strategies that are tailored for specific pathologic conditions. However, identifications of such differences have been difficult.

The present study examined, in rats, the expression of stress-related proteins to compare the RV and LV free walls at the basal level as well as in response to ischemia/reperfusion (I/R) injury in isolated hearts perfused through the biventricular working heart system. These experiments identified some differences between the two ventricles.

## Materials and Methods

### Animal treatment

Male Sprague-Dawley (SD) rats were housed in the animal care facility at Georgetown University Medical Center and were fed normal rat chow. Animals were anesthetized by the inhalation of isoflurane, the chest was opened and the heart and the lung were quickly excised. The RV and LV free walls were surgically dissected.

For I/R experiments, isolated hearts were perfused, on a non-recirculating Bi-Ventricular Isolated Working Heart Perfusion System (Harvard Apparatus, Holliston, MA, USA) with a modified Krebs–Henseleit bicarbonate buffer containing (in mM): 118 NaCl, 4.7 KCl, 1.7 CaCl2, 1.2 KH2PO4, 1.2 MgSO4, 25 NaHCO3, and 10 glucose, pH 7.4, aerated with a 95% O2/5% CO2 gas mixture at 37°C. The hearts were subjected to perfusion with Krebs-Henseleit buffer for 15 min and then to 30 min ischemia and 2 h reperfusion. The RV and LV free walls were then surgically dissected.

The Georgetown University Animal Care and Use Committee approved all animal experiments, and the investigation conformed to the National Institutes of Health Guide for the Care and Use of Laboratory Animals.

### Western blot analysis

RV and LV free wall tissues were homogenized with a Polytron and protein gel electrophoresis samples were prepared as previously described^6,7^. For Western blotting, equal protein amounts of samples were electrophoresed through a reducing SDS polyacrylamide gel and electroblotted onto a nitrocellulose membrane. The membrane was blocked and incubated with primary antibodies against platelet/endothelial cell adhesion molecule 1 (PECAM-1), vascular endothelial growth factor (VEGF), Bcl-xL, GATA4, tropomyosin, troponin T-C, sarco/endoplasmic reticulum Ca2+-ATPase (SERCA2), triadin, glyceraldehyde 3-phosphate dehydrogenase (G3PDH), β-actin (Santa Cruz Biotechnology, Inc. Dallas, TX, USA), microtubule-associated proteins 1A/1B light chain 3-A and -B (LC3A and LC3B), parkin, and cleaved caspase-3 (Cell Signaling Technology, Danvers, MA, USA). Levels of proteins were detected using horseradish peroxidase-linked secondary antibodies and Enhanced Chemiluminescence System (GE Healthcare Life Sciences, Pittsburgh, PA, USA).

### Statistical analysis

Means and standard errors of the means (SEM) were calculated. A two-tailed Student’s t test was used to analyze comparisons between two groups. P < 0.05 was considered significant.

## Results

**Figure 1:**
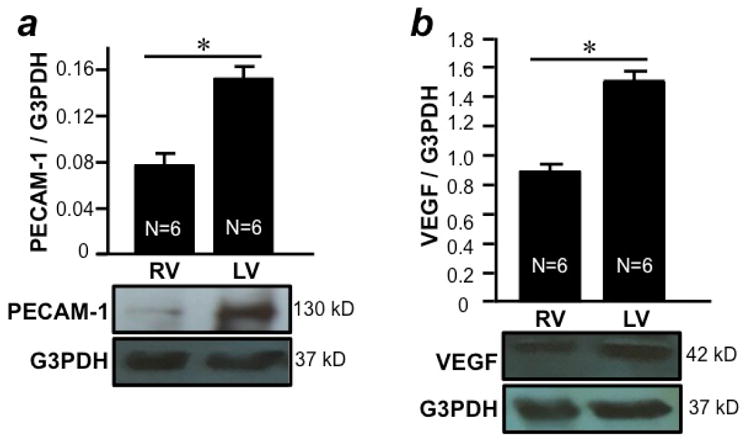
Differential expression of angiogenesis markers in the RV and LV free walls RV and LV free walls were isolated from male SD rats and homogenized. Homogenates were subjected to Western blotting to monitor the expression of (a) PECAM-1 and (b) VEGF proteins. Bar graphs represent means ± SEM of the ratio of PECAM-1 or VEGF to G3PDH. The symbol * denotes that RV and LV values are significantly different from each other at P<0.05.

To explore possible differences between the RV and LV in tolerance to stress, we first determine the levels of angiogenesis indicators. We found that the expression of VEGF, a major contributor in increasing the number of capillaries during angiogenesis, is significantly higher in the LV free wall compared to the RV free wall in healthy rat hearts (Figure 1A). Consistently, PECAM-1, a well-used marker for endothelial cells and angiogenesis, is more highly expressed in the LV free wall than in the RV free wall (Figure 1B). These results suggest that the LV is more vascularized compared to the RV.

**Figure 2:**
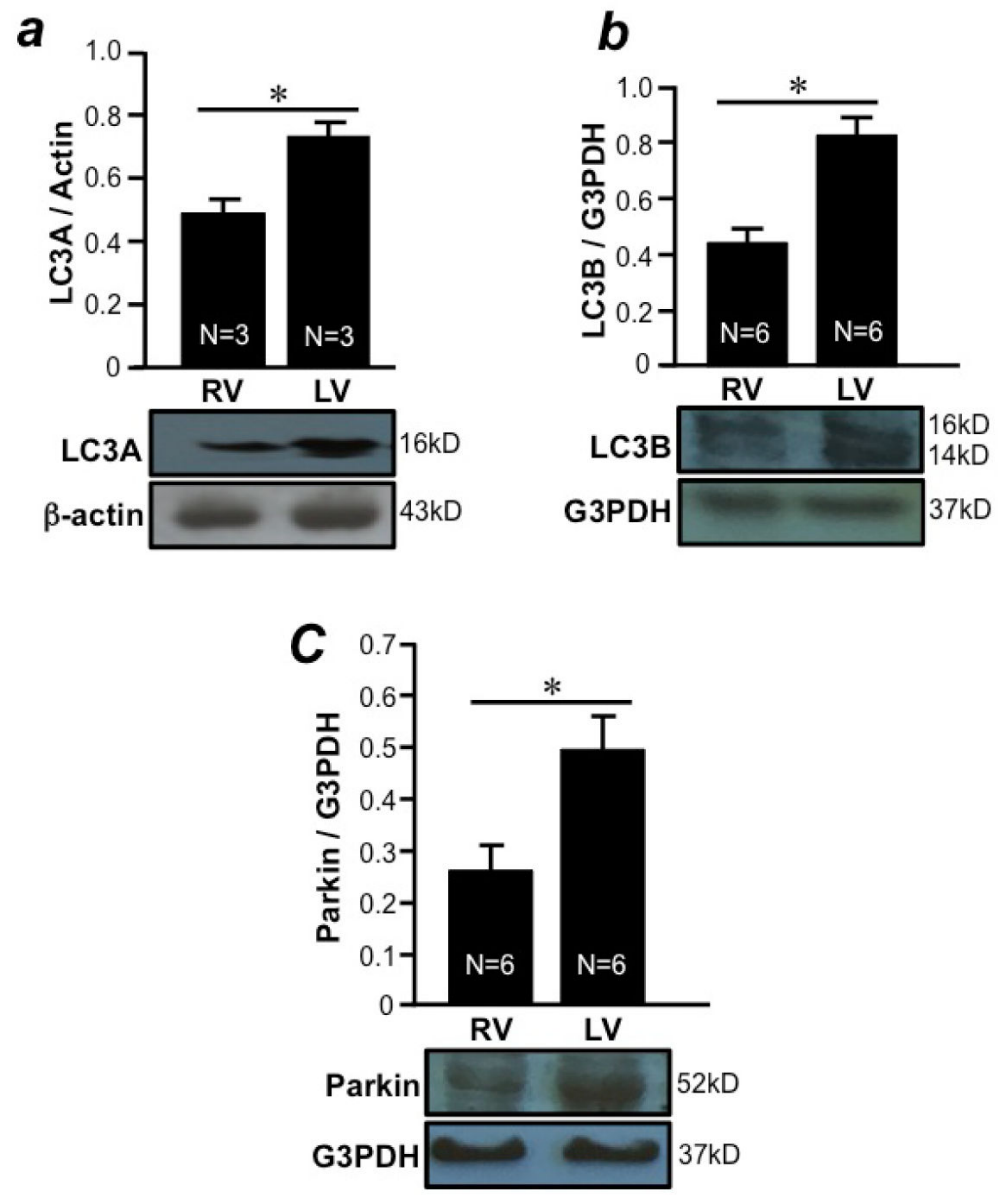
Differential expression of autophagy and mitophagy markers in the RV and LV free walls RV and LV free walls were isolated from male SD rats and homogenized. Homogenates were subjected to Western blotting to monitor the expression of (a) LC3A, (b) LC3B and (c) parkin proteins. Bar graphs represent means ± SEM of the ratio of proteins of interest to G3PDH. The symbol * denotes that RV and LV values are significantly different from each other at P<0.05.

Autophagy has recently gained considerable attention as a regulator of cell survival^8,9^. Light Chain 3 (LC3) molecules serve as key effectors for the promotion of autophagy. We found that both LC3A (Figure 2A) and LC3B (Figure 2B) isoforms were more highly expressed in the LV free wall compared to the RV free wall. Parkin, which mediates selective mitochondrial autophagy called mitophagy, was also found to be more highly expressed in the LV free wall compared to the RV free wall. Thus, the LV may be more protected against stress through the mechanisms of autophagy/mitophagy.

To determine the stress-mediated changes in the LV and RV, we utilized the biventricular working heart technique. In this system, isolated rat hearts are cannulated through the inferior vena cava and the pulmonary artery to perfuse the RV in the working mode and through the pulmonary vein and the aorta to perfuse the LV in the working mode. The coronary circulation is also perfused from the aorta. This system should simulate physiologically relevant situations for both the LV and RV. Using this system, we subjected the isolated rat heart to 30 min of no-flow ischemia and subsequent reperfusion.

**Figure 3:**
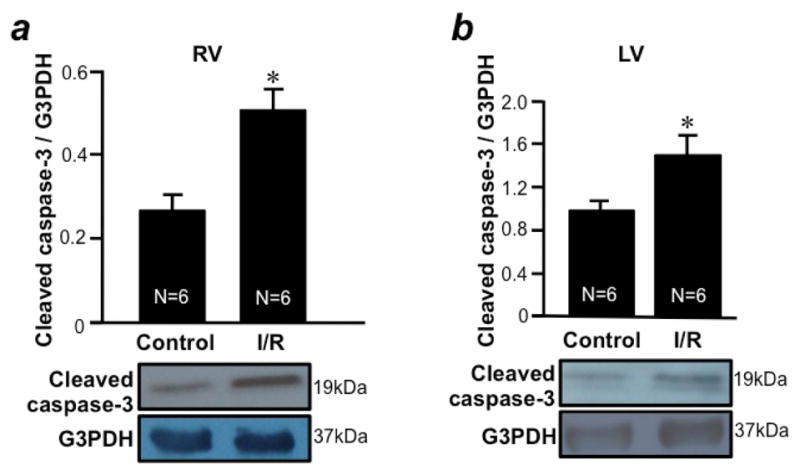
I/R promotes apoptosis in the RV and LV free walls Isolated rat hearts were perfused in a biventricular working heart mode and subjected to 30 min of global ischemia and 2 h of reperfusion. RV and LV free wall homogenates were subjected to Western blotting to monitor the expression of cleaved caspase-3 as a marker of apoptosis in the (a) RV and (b) LV. Bar graphs represent means ± SEM of the ratio of cleaved caspase-3 expression to that of G3PDH. The symbol * denotes that I/R values are significantly different from control values at P<0.05.

**Figure 4:**
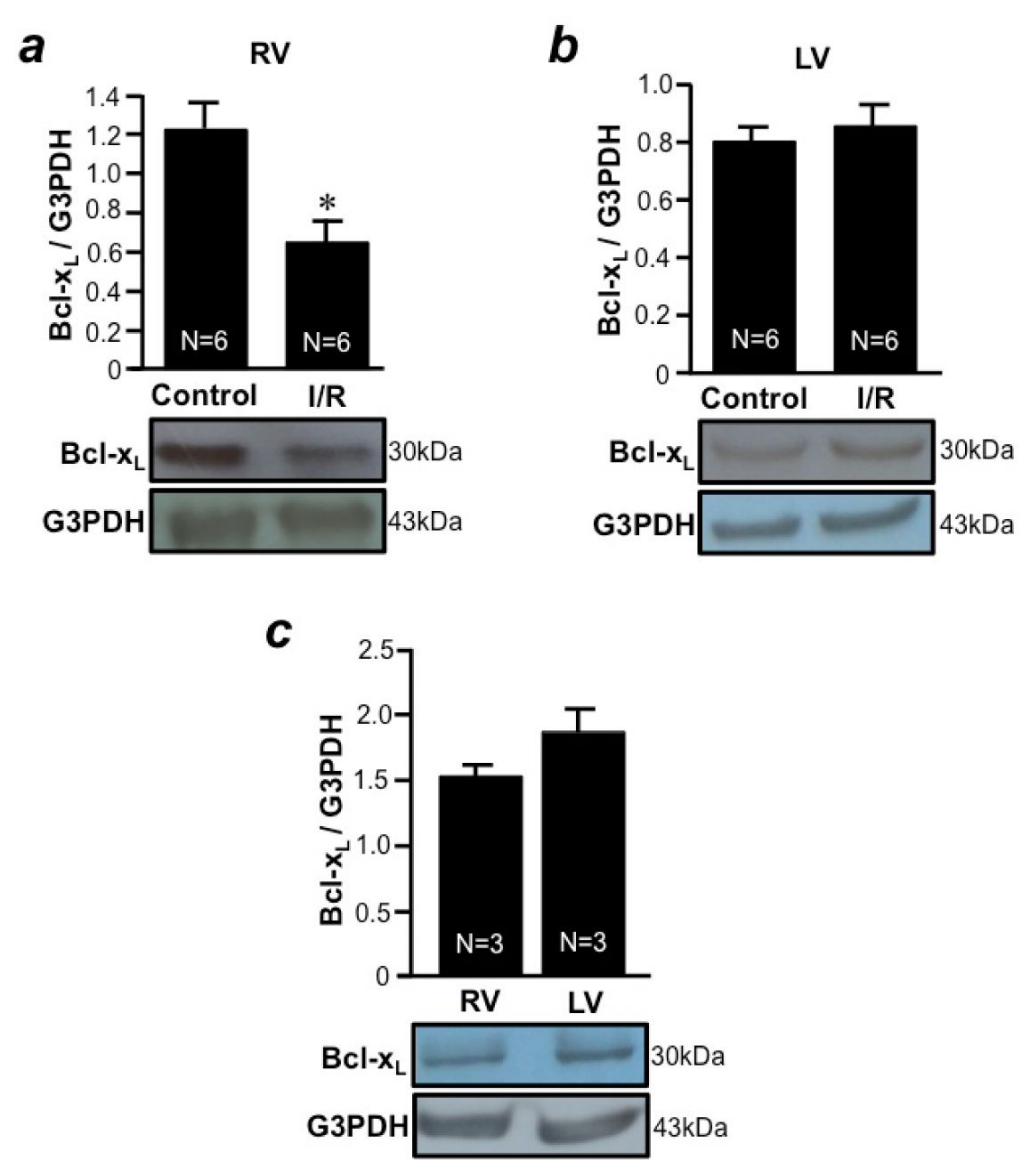
I/R downregulates Bcl-xL only in the RV, but not in the LV (A & B) Isolated rat hearts were perfused in a biventricular working heart mode and subjected to 30 min of global ischemia and 2 h of reperfusion. RV and LV free wall homogenates were subjected to Western blotting to monitor the expression of an anti-apoptotic protein, Bcl-xL, in the (a) RV and (b) LV. (c) RV and LV free wall homogenates were subjected to Western blotting to monitor the expression of Bcl-xL. Bar graphs represent means ± SEM of the ratio of Bcl-xL to G3PDH. The symbol * denotes that the I/R value is significantly different from the control value at P<0.05.

**Figure 5:**
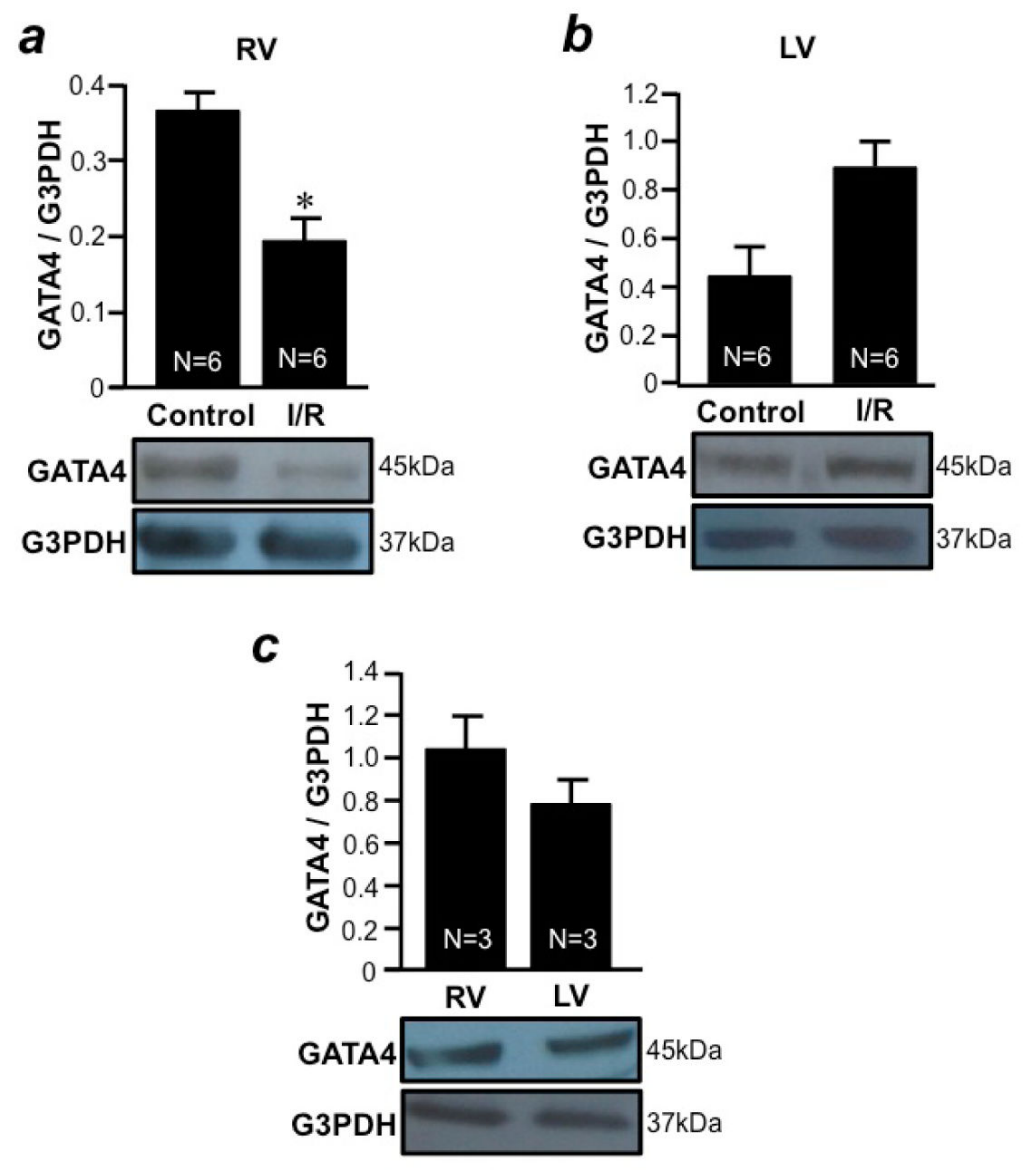
I/R downregulates GATA4 only in the RV, but not in the LV (a & b) Isolated rat hearts were perfused in a biventricular working heart mode and subjected to 30 min of global ischemia and 2 h of reperfusion. RV and LV free wall homogenates were subjected to Western blotting to monitor the expression of GATA4 (a transcription factor that regulates Bcl-xL expression) in the (a) RV and (b) LV. (c) RV and LV free wall homogenates were subjected to Western blotting to monitor the expression of GATA4. Bar graphs represent means ± SEM of the ratio of GATA4 to G3PDH. The symbol * denotes that the I/R value is significantly different from the control value at P<0.05.

I/R was found to promote apoptosis in both RV (Figure 3A) and LV (Figure 3B) free walls as determined by monitoring the level of cleaved caspase-3. The downregulation of anti-apoptotic protein should promote apoptosis. Thus, we monitored the expression level of Bcl-xL. We found that, in response to I/R, Bcl-xL was downregulated only in the RV free wall (Figure 4A), but not in the LV free wall (Figure 4B). The basal expression levels of Bcl-xL in the RV and LV free walls were comparable (Figure 4C). GATA4 is a key transcriptional regulator of Bcl-xL in the heart^10^. I/R was found to downregulate GATA4 only in the RV free wall (Figure 5A), but not in the LV free wall (Figure 5B). The basal expression levels of GATA4 in RV and LV free walls were found to be comparable (Figure 5C). These results suggest that, while both RV and LV free walls are susceptible to apoptosis in response to I/R, these two ventricles utilize distinct mechanisms; i.e. only the RV may use the mechanism of apoptosis that involves GATA4- dependent Bcl-xL downregulation.

**Figure 6:**
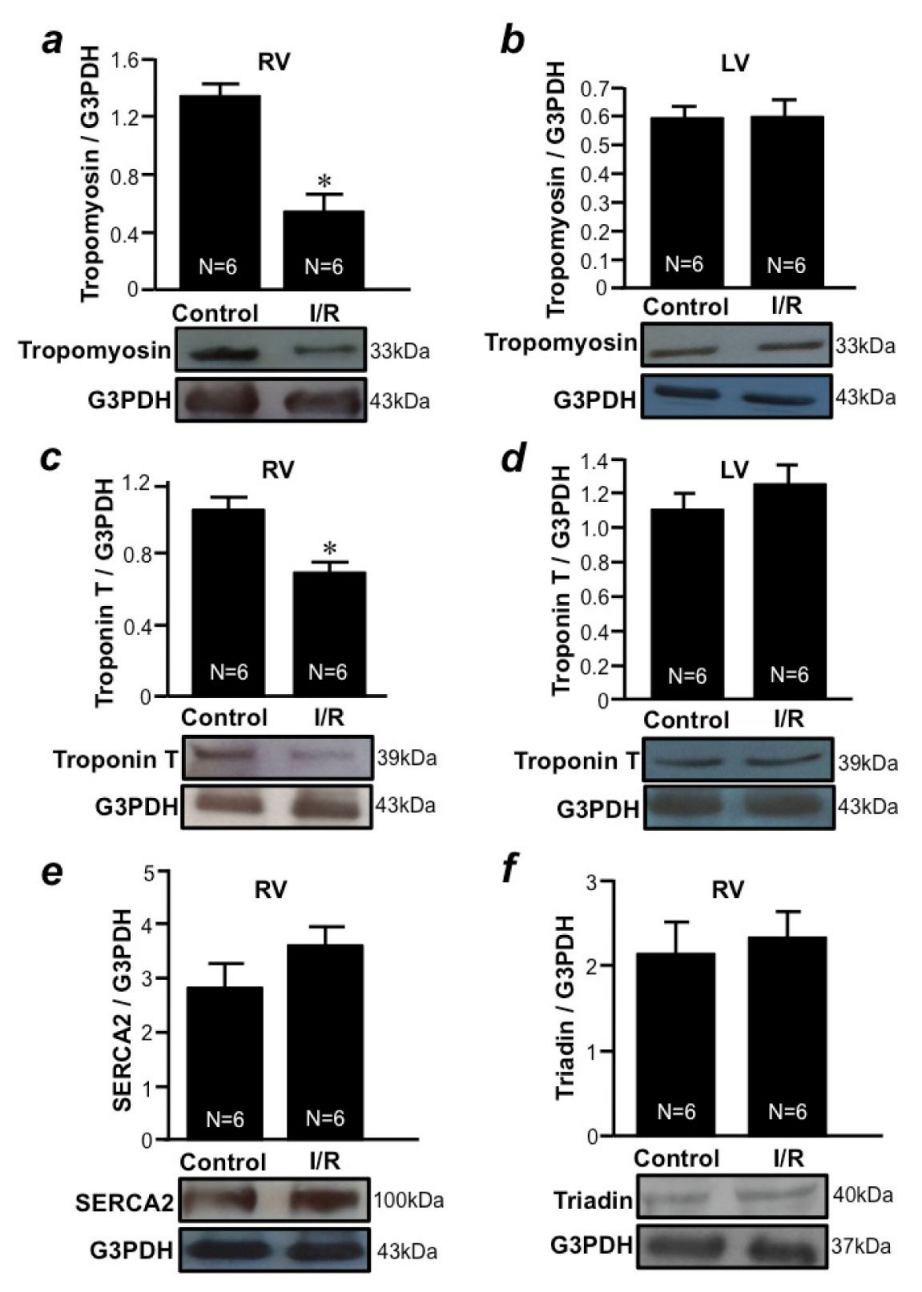
I/R downregulates tropomyosin and troponin T only in the RV, but not in the LV Isolated rat hearts were perfused in a biventricular working heart mode and subjected to 30 min of global ischemia and 2 h of reperfusion. RV and LV free wall homogenates were subjected to Western blotting to monitor the expression of (a & b) tropomyosin, (c & d) troponin T, (e) SERCA2 and (f) triadin in the (a, c, e & f) RV and (b & d) LV. Bar graphs represent means ± SEM. The symbol * denotes that the I/R values are significantly different from the control values at P<0.05.

During cardiac damage, some muscle contractile proteins are released from the heart. We found that the level of tropomyosin (Figure 6A) was decreased by I/R only in the RV free wall, but not in LV free wall (Figure 6B). Similarly, I/R caused the downregulation of troponin T only in the RV free wall (Figure 6C), but not in the LV free wall (Figure 6D). The expression levels of some membrane bound proteins such as SERCA2 (Figure 6E) and triadin (Figure 6F) were not altered by I/R in the RV free wall. These results demonstrate that the RV is more susceptible to cardiac damage, specifically the downregulation of contractile proteins, in response to I/R compared to the LV.

## Discussion

Information about the difference between the mechanisms of how the RV and LV respond to and cope with stress such as I/R is not currently available. The present study provided important information on the differences between the two ventricles in adult rats. First, the LV free wall exhibits higher levels of endothelial and angiogenesis markers compared to the RV free wall, which reflect the need of the LV to exert higher contractile force. The LV also has increased levels of proteins that are involved in autophagy such as LC3 isoforms as well as in mitophagy such as parkin. Autophagy and mitophagy have been implicated in providing cell survival mechanisms in the heart^8,9^, thus the LV may possess stronger protective mechanisms against stress. To test this hypothesis, we subjected the isolated rat heart to biventricular working heart perfusion.

Unlike the Langendorff isolated heart model that provides the retrograde perfusion of the coronary artery only, the working heart perfusion mode allows for more closely simulating the biology of the heart with ventricular filling via atria and the normal direction of the flow through the aorta. The working heart preparation has widely been used to study the LV in the condition, in which only the pulmonary vein and aorta are cannulated while the RV chambers are not filled. In the preset study, since the inferior vena cava and the pulmonary artery are also cannulated, the right atrium and RV are also perfused in the working mode. Subjecting the heart, in this perfusion system, to global ischemia for 30 min followed by reperfusion resulted in the increased levels of cleaved caspase-3 as an indication of apoptosis in both RV and LV free walls in a similar fashion.

Mechanisms of I/R-induced myocardial apoptosis appear to be different between the RV and LV. The downregulation of anti-apoptotic Bcl-xL that can lead to apoptosis occurred only in the RV, but not in the LV. We have previously shown that GATA4 is a regulator of Bcl-xL gene transcription and that apoptotic stimuli downregulate Bcl-xL via inhibiting GATA4 gene expression in cardiac myocytes^10^. The present study revealed that I/R downregulated the GATA4 expression in the RV but not in the LV, consistent with the idea that I/R specifically activates the GATA4/Bcl-xL pathway in the RV.

The hypothesis that the LV is more resistant to cope with stress is supported by our results showing that the expression of contractile proteins such as tropomyosin and troponin-T are reduced in response to I/R only in the RV, but not in the LV. These cytosolic proteins leak out of the cells during stress, and our results indicate that I/R caused the sarcolemmal leakage only in the RV.

The RV and LV free walls are originated from different precursors^4^. During development, the existence of differential mechanisms for the formation of RV and LV with the chamber-specific expression of regulatory proteins has been well documented^4^. However, after birth, terminally differentiated cardiomyocytes loose the differential expression patterns and the expression profile of the RV and LV of adult hearts are remarkably similar. We were unable to detect differences between RV and LV free wall protein expression patterns of the adult rat hearts using two-dimensional gel electrophoresis (unpublished results). However, we have previously identified that the basal expression of the CBF/NF-Y transcription factor is higher in the RV compared to the LV^7^ and that the monoamine oxidase A is lower in the RV compared to the LV^11^ in rats. The present study further revealed five more proteins that are differentially expressed in the adult RV and LV free walls.

## Conclusions

The RV and the LV free walls possess distinct response mechanisms against stress. Specifically, suppression of the GATA4/Bcl-xL pathway and dowregulation of tropomyosin and troponin T are RV-specific events that occur in response to stress. Identifications of fine differences between the RV and LV free walls should help developing chamber-specific therapeutic strategies to treat cardiovascular diseases, in particular right heart failure that occurs in pulmonary hypertension.

## List of Abbreviations

G3PDH: glyceraldehyde 3-phosphate dehydrogenase
I/R: ischemia/reperfusion
LC3A: microtubule-associated proteins 1A/1B light chain 3-A
LC3B: microtubule-associated proteins 1A/1B light chain 3-B
LV: left ventricle
PECAM-1: platelet/endothelial cell adhesion molecule 1
RV: right ventricle
SD: Sprague-Dawley
SEM: standard errors of the means
SERCA2: sarco/endoplasmic reticulum Ca2+-ATPase
VEGF: vascular endothelial growth factor

## References

[1] Voelkel NF, Quaife RA, Leinwand LA, Barst RJ, McGoon MD, Meldrum DR (2006). Right ventricular function and failure: The need to know more. Report of a National Heart, Lung and Blood Institute Working Group on Cellular and Molecular Mechanisms of Right Heart Failure. Circulation.

[2] Boxt LM (1999). Radiology of the right ventricle. Radiol Clin North Am.

[3] Budev MM, Arroliga AC, Wiedemann HP, Matthay RA (2003). Cor pulmonale: an overview. Semin Respir Crit Care Med.

[4] Srivastava D (2006). Making or breaking the heart: from lineage determination to morphogenesis. Cell.

[5] Suzuki YJ, Yuan JXJ, Garcia JGN, Hales CA, Archer SL, Rich S, West JB (2011). Molecular basis of right ventricular hypertrophy and failure in pulmonary vascular disease. Textbook of Pulmonary Vascular Disease.

[6] Park A, Suzuki YJ (2007). Effects of intermittent hypoxia on oxidative stress-induced myocardial damage in mice. J Appl Physiol.

[7] Park A, Wong C, Jelinkova L, Liu L, Nagase H, Suzuki YJ (2010). Pulmonary hypertension-induced GATA4 activation in the right ventricle. Hypertension.

[8] Moyzis AG, Sadoshima J, Gustafsson ÅB (2015). Mending a broken heart: the role of mitophagy in cardioprotection. Am J Physiol Heart Circ Physiol.

[9] Hamacher-Brady A, Brady NR, Gottlieb RA (2006). Enhancing macroautophagy protects against ischemia/reperfusion injury in cardiac myocytes. J Biol Chem.

[10] Kitta K, Day RM, Kim Y, Torregroza I, Evans T, Suzuki YJ (2003). Hepatocyte growth factor induces GATA-4 phosphorylation and cell survival in cardiac muscle cells. J Biol Chem.

[11] Liu L, Marcocci L, Wong CM, Park AM, Suzuki YJ (2008). Serotonin-mediated protein carbonylation in the right heart. Free Radic Biol Med.

